# Significance of Morphology in Characterizing Human Health Risk from di(2-ethylhexyl) Phthalate in Polyvinyl Chloride Microplastics in Groundwater

**DOI:** 10.3390/toxics13020105

**Published:** 2025-01-28

**Authors:** Ki-Han Song, Sang-Gyu Yoon, Jin-Yong Lee, Jinsung An

**Affiliations:** 1 Department of Civil Engineering, Seoul National University of Science and Technology, Seoul 01811, Republic of Korea; kihansong@seoultech.ac.kr; 2 Department of Smart City Engineering, Hanyang University ERICA, Ansan 15588, Republic of Korea; sgyoon95@hanyang.ac.kr; 3 Department of Geology, Kangwon National University, Chuncheon 24341, Republic of Korea; hydrolee@kangwon.ac.kr; 4 Department of Civil & Environmental Engineering, Hanyang University ERICA, Ansan 15588, Republic of Korea

**Keywords:** microplastics, additives, probabilistic risk assessment, Monte Carlo simulation, probability distribution

## Abstract

In this study, a human health risk assessment was performed on the ingestion route of groundwater containing polyvinyl chloride (PVC) microplastics (MPs), and the carcinogenic and non-carcinogenic risks of di(2-ethylhexyl) phthalate (DEHP), a representative additive, were determined. In particular, the impact of volume diversity according to the shape (morphology) of PVC MP (fragment, fiber, film) on the risk characterization was intensively explored. Firstly, a continuous particle size distribution following a power function was derived using the abundance ratio of PVC MPs by size in the investigated groundwater, and human health risk assessment for DEHP in the PVC MPs was performed through the volume distribution according to the shape of MPs. DEHP human health risk assessment showed an excess cancer risk (ECR) of below 10^−6^ for a 95% cumulative probability for all MP shapes, but the values varied depending on the shape. Sensitivity analysis showed that the parameter that most affected human health risk was MP volume, second to concentration, which is dependent on MP shape. Therefore, it is necessary to consider the variety of MP shapes during human health risk assessment, and it can be achieved through probabilistic risk assessment utilizing the probability distribution for size and shape of MPs.

## 1. Introduction

Plastics are widely used in our daily lives due to their durability, lightness, stability, and low costs [1]. As a result, plastic demand and production have increased significantly. Annual global plastic production increased from 1,300,000 tons in 1950 to 359,000,000 tons in 2018 and is expected to exceed approximately 500,000,000 tons in 2025 [2]. However, inadequate management and treatment practices have resulted in the inflow and accumulation of plastics in the aquatic and soil environments, causing significant environmental problems [2,3,4]. In addition, the accumulated plastic in the environment fragments due to physical, chemical, and biological weathering processes [5]. Microplastics (MPs), less than 5 mm in size, are formed through the disintegration of regular plastics into smaller pieces and have become a worldwide concern [6,7,8].

Groundwater is a freshwater source for more than two billion people worldwide [9]. Several studies have reported the adverse impact of MPs on surface water and marine and soil environments [10,11,12,13], but studies on the impact of MPs on groundwater are limited [14,15]. However, recent studies have reported that MPs can enter groundwater through leaching from heavy rains and irrigation, vertical flow via soil biological activity (e.g., burrows, adherence to organisms), seawater intrusion, and other routes, including the exchange of surface and groundwater near and downstream from rivers and streams [13,14,15,16,17,18,19,20,21]. Humans are at risk of continuous exposure to MPs through water and food contaminated with MPs [22,23,24]. Since groundwater accounts for about 25% of drinking water worldwide [25], the exposure to groundwater MPs in humans is estimated to be high. However, research on the impact of this exposure on humans and their health is currently lacking [23].

Additives in plastics, including plasticizers, antioxidants, and dyes, are used to improve their physicochemical properties [26]. These additives do not combine with monomers strongly and hence can be easily released and discharged into the environment, becoming toxic to humans and ecosystems [5]. In particular, di(2-ethylhexyl) phthalate (DEHP) is the most widely used plasticizer worldwide, accounting for approximately 40% of the global plasticizer market [27], and is an endocrine disruptor with potential carcinogenic and noncarcinogenic risks [28]. Owing to its high compatibility with polyvinyl chloride (PVC), DEHP is most frequently used in PVC-containing products [29]. PVC can contain up to about 30–35% DEHP [30]. PVC is widely used in manufacturing a variety of consumer products, including packaging and toys, with an estimated annual production of approximately 3 million tons worldwide [31]. PVC can be categorized into rigid PVC and flexible PVC, with flexible PVC using higher amounts of DEHP (approximately 60%) [32]. Therefore, the inflow and accumulation of PVC MPs in groundwater, and the intake of DEHP leached into groundwater as well as the PVC MP particles themselves, may potentially have adverse impacts on human health.

In this regard, risk assessments have been performed in many reported studies to evaluate the potential impacts of MP-containing additives in the environment on ecosystems and human health [33,34,35]. Most of the previously reported ecological and human risk assessments of MP-containing additives did not sufficiently reflect the diversity of MPs’ characteristics (e.g., size, shape (morphology)) and were performed based on individual characteristics or average values [33,34,35]. The characteristics of MPs in the actual environment change continuously due to physicochemical weathering processes, and these changes lead to a decrease in MP particle size (i.e., increase in the continuity of particle size), changes in shape (i.e., increase in atypical MP such as fragment type), and subsequent changes in additive release [36]. As a result, the diversity of MPs’ characteristics can act as a major variable in the ecological and human risk assessment of MP-containing additives. Therefore, the existing approach has limitations in properly assessing the diversity of MPs in the actual environment and the resulting uncertainty and not only lowers the reliability of the risk assessment results, but also makes it difficult to compare results among researchers or perform integrated interpretations [6,37,38,39]. Recent studies have attempted to overcome these limitations by introducing a probabilistic approach to express the various characteristics of MPs as probability distributions and quantitatively evaluate their risk through this [6,37,38,39]. Kooi et al. [38] effectively reflected the variability and diversity of MP characteristics in real environments, which are difficult to capture with individual and discontinuous values, by expressing the characteristics of MPs (i.e., the diversity of size) as continuous probability distributions. This approach overcomes the limitations of existing MP risk assessment approaches and provides a basis for more realistic risk assessment.

In this study, we assessed the potential impact of DEHP on human health through the intake of groundwater containing DEHP released from PVC MPs as well as of PVC MP particles themselves (i.e., DEHP released from PVC MPs during digestion processes). In particular, the effect of diversity in MP properties (i.e., volume according to the morphology) on human health risk characterization was quantified using continuous probability distribution following the power law function and a Monte Carlo simulation. Sensitivity analysis was used to investigate the factors that mostly affect the human health risk for DEHP in PVC MPs. This study can provide meaningful insights into the health of humans likely to be exposed to MPs contained in groundwater through systematic risk characterization, and it also emphasizes the need for identification of specific properties of MPs.

## 2. Materials and Methods

### 2.1. Leaching Experiment on DEHP Contained in PVC MPs

To investigate the leaching amount of DEHP in PVC MPs, leaching experiments were conducted using PVC MPs containing 34.1% (*w*/*w*) DEHP and artificial groundwater. Details of the artificial groundwater composition are provided in Table S1. In the experiments, 0.2 g of PVC MPs (particle size: <125 μm) and 40 mL of artificial groundwater were placed in a glass vial and stirred at 100 rpm for 48 h using an end-over-end shaker. After stirring, the mixture was filtered through a 0.45 μm filter, and the concentration of DEHP in the filtrate was analyzed using gas chromatography–mass spectrometry (GC-MS, Agilent 7890B, Santa Clara, CA, USA) (Table S2). The amount of DEHP leached into the artificial groundwater was approximately 4.37 ± 2.30 µg/g. This amount corresponds to approximately 0.0013% of the total weight of DEHP contained in the MPs. The calculated DEHP leaching rate was used as an input parameter for the probabilistic human health risk assessment utilizing a Monte Carlo simulation.

### 2.2. Determination of Volume Probability Distribution Based on MP Size and Shape

MP mass for determining DEHP exposure concentration (C_w_) was calculated by multiplying MPs’ volume with their density (ρ·V) (Equations (1)–(3)), and each parameter may have a significant impact on the DEHP exposure concentration.

(1) $$C_{w}={\;C}_{w1}+{\;C}_{w2}$$

(2) $$C_{w1}={\;C}_{\mathrm{mp}}\;\cdot \left(\rho V\right)\;\cdot \;R\;\cdot {\;L}_{1}$$

(3) $$C_{w2}={\;C}_{\mathrm{mp}}\;\cdot \left(\rho V\right)\;\cdot \;R\;\cdot {\;L}_{2}$$

where C_w_, C_w1_, and C_w2_ refer to the total DEHP concentration exposed to the human body by oral exposure to groundwater, DEHP concentration leached from MPs into groundwater, and DEHP concentration leached from MPs inside the human body (i.e., the concentration of DEHP leached from MP into digestive juice during the digestion process after ingestion of groundwater containing MP), respectively. R is the DEHP content in PVC MPs. L_1_ is the percentage of DEHP leached from MPs into groundwater based on MP weight obtained from Section 2.1. L_2_ is the percentage of DEHP that can be leached from MPs into the human body based on MP weight obtained from the previous studies.

Even if MPs’ particle size (diameter) is the same, the MPs’ volume may differ depending on their shape. In other words, the DEHP exposure concentration (C_w_) can be calculated differently according to the MPs’ shape. In general, MPs found in the environment (e.g., seawater, freshwater, and sediments) can be categorized into fragments, fibers, films, and spheres, based on their shape [40], and disregarding the diversity of the MPs shape could lead to significant uncertainty in MP risk assessment. To take these aspects into account, a probability distribution of the volume of MPs for each shape (fragment, fiber, film) of MP was obtained for the probabilistic human health risk assessment that considered the diversity of MP shapes in this study.

First, the size of the MPs in the investigated groundwater (Table S3) was presented as a series of continuous empirical probability distributions that follow a power law distribution. The properties of MPs in various environmental media are known to follow continuous distribution [6], and the particle size distribution of MPs is reported to follow continuous power law distribution [3,38]. This was attributed to the continuous generation of MPs in environmental media due to the fragmentation of raw plastics. Although knowledge on the process of MP fragmentation in groundwater is limited, it has been documented that the smaller the MP particle size, the easier it is to reach groundwater [41]. Therefore, it is reasonable for MPs in groundwater to exhibit a particle size distribution in which the smaller the size, the larger the abundance ratio.

A power function curve (Equations (4) and (5)) was applied to the MP size data obtained to determine α (power law index) through data fitting, and the compatibility was represented by the coefficient of determination (R^2^).

(4) $$y\;=\;b\cdot x^{-\alpha }$$

(5) $$b\;=\left(\alpha -1\right)\cdot x_{\min}^{\left(\alpha -1\right)}$$

where y refers to the abundance ratio (%) of MPs, x represents the particle size of the largest MP (μm), and α and b are the fitting parameters. The value of b varies with y, and it can be represented as a function of x_min_ (minimum particle size) and α.

The volume (cm^3^) of the MPs was determined; fragment, fiber, and film were considered as ellipses (4πabc/3; a,b,c: radii in each direction), cylinders (πr^2^H), and hexahedrons, respectively, for volume determination using a continuous probability distribution for the size of MPs obtained. The upper and lower values of the general length:width:height (L:W:H) ratio for each MP shape (fragment, sphere, fiber, and film) used to determine the volume of each MP shape are shown in Table S4 [38].

### 2.3. Probabilistic Human Health Risk Assessment of DEHP Contained in MPs in Groundwater

The probabilistic human health risk assessment of the DEHP contained in PVC MPs in groundwater was performed, and concurrently the impact of the uncertainty caused by the PVC MP particle properties diversity (volume according to the morphology) was investigated. The hazard quotient (HQ) for the non-carcinogenic risk characterization and the excess cancer risk (ECR) for the carcinogenic risk characterization were determined by considering the route of oral ingestion of groundwater containing PVC MPs according to Equations (6)–(8) [42,43].

(6) $$\mathrm{ECR}\;={\;\mathrm{SF}}_{0}\;\cdot \;\mathrm{ADD}$$

(7) $$\mathrm{HQ}\;=\frac{\mathrm{ADD}}{{\mathrm{RfD}}_{0}}$$

(8) $$\mathrm{ADD}\;\left(\mathrm{mg}/\mathrm{kg}-\mathrm{day}\right)=\frac{C_{w}\;\cdot {\;\mathrm{CR}}_{w}\;\cdot \;\mathrm{EF}\;\cdot \;\mathrm{ED}}{\mathrm{BW}\;\cdot \;\mathrm{AT}}$$

The HQ and ECR were determined with the aid of the Monte Carlo simulation (Oracle Crystal Ball software ver. 11.1.3.0.0.) and were presented through the 95th percentiles. SF_0_ and RfD_0_ refer to the slope factor for carcinogenesis ((mg/kg-day)^−1^) and oral reference dose (mg/kg-day), respectively, and these values were obtained from the United States Environmental Protection Agency (USEPA) Integrated Risk Information System [42]. The DEHP exposure concentration through the ingestion of groundwater (C_w_) can vary significantly based on the number of MPs studied (C_mp_), density (ρ), volume (V), DEHP content in MPs (R), and the leaching rate (L_1_, L_2_) according to Equations (1)–(3). In the case of C_mp_, the mode, minimum value, and maximum value were assumed to follow a triangular distribution at 0, 0, and 79.23, respectively, which was ascertained through literature review [18,44,45] using the PVC MP concentration (Table S3) in the groundwater. In the case of ρ, a density range (density range of PVC MPs commonly found in the environment: 1.10–1.58 mg/cm^3^) that was ascertained through literature review [38] was applied. In case of V, the MP volume probability distribution following the power law derived in Section 2.2 was used. In the case of R, DEHP content in MPs (Table S5), which was ascertained through literature review [30,44,46,47,48,49,50], based on minimum extreme distribution (mode: 0.29, scale: 0.08), was applied. Detailed information about the parameters used in the Monte Carlo simulation are presented in Table 1.

A sensitivity analysis was performed to ascertain the significant parameters that impact the rate of carcinogenic and non-carcinogenic risks based on the route of ingestion of DEHP-containing MPs in groundwater.

## 3. Results and Discussion

### 3.1. MP Size and Shape-Specific Volume Probability Distribution

**Figure 1:**
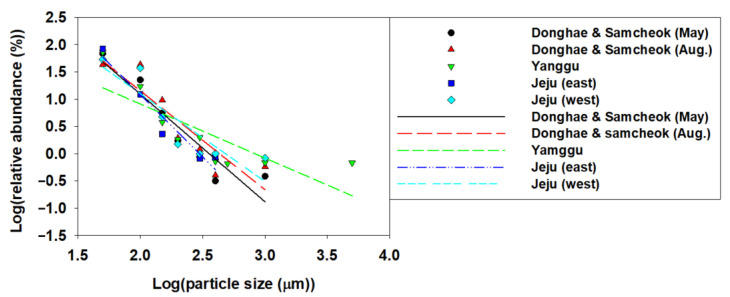
Relative abundance (%) according to diverse MP particle size in groundwater presented in a log–log scale. The relative abundance data for each MP size sampled from five sampling sites obtained from the previous studies [18,44,45] were used, and the determined α values in Donghae and Samcheok (May), Donghae and Samcheok (Aug), Yanggu, East Jeju, and West Jeju were 1.97, 1.81, 0.99, 2.28, and 1.62, respectively, and the average value was 1.74 ± 0.48 (fitting rates (R^2^) for each site, Donghae and Samcheok (May), Donghae and Samcheok (Aug), Yanggu, East Jeju, and West Jeju, were 0.8783, 0.8172, 0.6559, 0.938, and 0.7848, respectively).

A single empirical equation (power function) was derived using the abundance ratio for each MP from the various MP sizes (μm) in groundwater in South Korea, ascertained from the literature review [18,44,45]. From the fitting results, the α value was determined according to the sampling location (Figure 1). In theory, the α value of the power function quotient is known to have a value between 0–3 [6], while the α values obtained in this study were 1.97 for Donghae and Samcheok (May), 1.81 for Donghae and Samcheok (Aug), 0.99 for Yanggu, 2.28 for East Jeju, and 1.62, for West Jeju, respectively, and their average value was 1.74 ± 0.48. In addition, the coefficient of determination (R^2^) ranged between 0.6559 and 0.9387. From the average α value obtained from the observed particle size information of MPs in Korean groundwater, a continuous particle size distribution of 20–5000 μm could be generated for MPs, and the generated particle size distribution and cumulative probability distribution are presented in Figure S1. The particle size distribution revealed a relatively higher abundance of smaller particles compared to larger ones.

To calculate the volume of each MP shape, the MP particle size probability distribution following the power law distribution was used. Hence, the MP volume probability distribution that followed the power law distribution was generated. The three probability distributions, generated based on the maximum value of the L:W:H ratio of MP shapes (i.e., fragment, fiber, and film), were applied to determine of the human health risk of DEHP in MPs in groundwater. The volume distribution of the sphere was not calculated because the maximum L:W:H ratio was equal to that of the fragment.

### 3.2. Probabilistic Human Health Risk Assessment of DEHP from MPs in Groundwater

**Figure 2:**
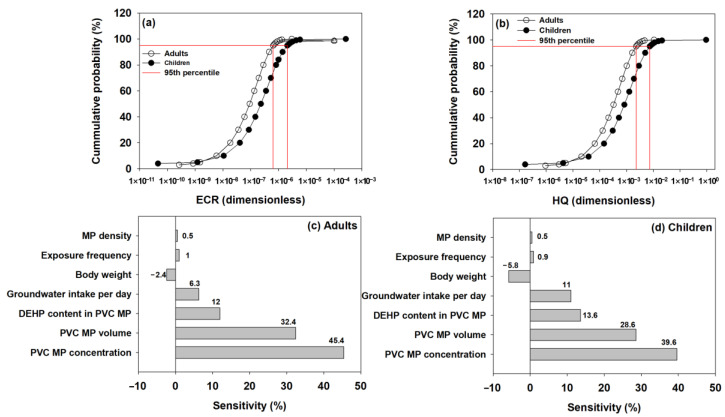
Cumulative probability distribution where excess cancer risk (ECR) and hazard quotient (HQ) were predicted for the di(2-ethylhexyl) phthalate (DEHP) in PVC MPs, based on the intake of groundwater contaminated with PVC MPs, presented on a log scale ((**a**): ERC, (**b**): HQ). HQ for adults and children did not exceed 1. ECR for adults and children at 95% cumulative probability was 6.4 × 10^−7^ and 2.1 × 10^−6^, respectively. Sensitivity analysis for adults (**c**) and children (**d**) are shown. The probability and sensitivity analyses were repeated 50,000 times during the Monte Carlo simulation (MP volume: uniform distribution).

The probabilistic human health risk of DEHP leached from PVC MPs in groundwater was assessed. Specifically, in the Monte Carlo simulation, the volume of PVC MPs was modeled using a uniform distribution. The volume of fiber-shaped PVC MPs, which had the smallest volume, was set as the minimum value, and the volume of fragment-shaped PVC MPs, which had the largest volume, was set as the maximum value. The estimated ECR and HQ values for DEHP under these conditions are presented in Figure 2. For both adults and children, HQ was less than 1 after assessing the non-carcinogenic risks. With respect to the cumulative probability of 95%, HQ for adults and children was less than 2.3 × 10^−3^ and 7.4 × 10^−3^, respectively (Figure 2b). This indicates that potential non-carcinogenic risk does not exist for DEHP exposure through the intake of MP-contaminated groundwater. In the meantime, ECR for adults and children was 6.4 × 10^−7^ and 2.1 × 10^−6^, respectively, for a 95% cumulative probability for the carcinogenic risk, and the probability that ECR would exceed 10^−6^ was found to be 1.4% and 15.9% for adults and children, respectively (Figure 2a). This indicates that DEHP exposure through the ingestion of MP-contaminated groundwater does not represent a negligible carcinogenic risk (the permissible ECR threshold value for regulation purposes is 1 × 10^−6^ to 1 × 10^−4^ [54]). However, there is a concern that the risk assessment results have been overestimated due to using a uniform distribution for the MP volumes in the groundwater. As mentioned earlier, the PVC MP mass for determining the DEHP exposure concentration (C_w_) was calculated by multiplying the volume and density of PVC MPs (ρ·V). As the volume of PVC MPs increases, their mass also increases, along with an increase in C_w_, ECR, and HQ. It is expected that the volume of MPs in groundwater follows a power law distribution. The proportion of smaller MP particles in the groundwater may be greater than the proportion of larger MP particles. Thus, the estimated PVC MP mass could be higher because of the application of uniform distribution (MP volume) during the determination of the DEHP exposure concentration. Nevertheless, this study conservatively conducted a human health risk assessment based on various reasonable assumptions based on the actual number of MP particles found in groundwater at various locations in South Korea, and the results showed that the carcinogenic and non-carcinogenic risks were at a worrisome level.

Sensitivity analysis was performed for only the ECR analysis results. The sensitivity analysis was conducted using Monte Carlo simulations to evaluate how variability in each parameter influenced the overall risk assessment. The parameters that largely impacted potential carcinogenic risks in adults and children were, in order, MP concentration, MP volume in groundwater, and DEHP content in MPs (Figure 2c,d). The parameter with the second largest impact on the potential carcinogenic risk was the volume of MPs in groundwater (adults: 32.4%; children: 28.6%). Through the sensitivity analysis, it was ascertained that the shape of MPs in groundwater may have impacted the potential carcinogenic risk assessment. The volume of MPs was calculated using shape-specific dimensions (L:W:H ratios), and different shapes (e.g., fragment, fiber, film) result in variations in surface area-to-volume ratios. Therefore, it is presumed that the shape of MPs indirectly impacted the potential carcinogenic risk assessment through its effect on MP volume.

### 3.3. Probabilistic Human Health Risk Assessment for Each MP Shape

**Figure 3:**
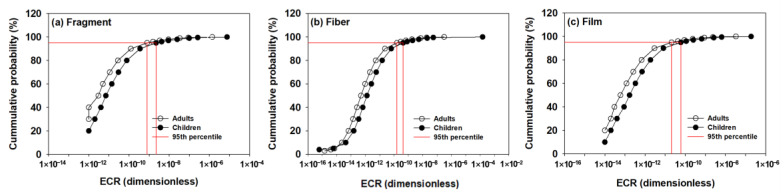
ECR assessment results for each MP shape ((**a**) fragment, (**b**) fiber, and (**c**) film) are presented in the log scale. ECR for adults at 95% cumulative probability for the fragment, fiber, and film shapes were 7.9 × 10^−10^, 1.6 × 10^−10^, and 2.1 × 10^−11^, respectively, and 2.2 × 10^−9^, 6.1 × 10^−11^, and 4.3 × 10^−10^, respectively, for children. Analysis results were obtained using a Monte Carlo simulation that was repeated 50,000 times.

The human health risk assessment applying the probability distribution for each MP shape (fragment, fiber, and film) was performed. The carcinogenic risk assessment results for each MP shape are shown in Figure 3. For the fragment shape, the ECR for adults and children at 95% cumulative probability were 7.9 × 10^−10^ and 2.2 × 10^−9^, respectively. In the case of the fiber shape, the ECR for adults and children at 95% cumulative probability was 1.6 × 10^−10^ and 4.3 × 10^−10^, respectively. In the case of the film shape, ECR for adults and children at 95% cumulative probability was 2.1 × 10^−11^ and 6.1 × 10^−11^, respectively. The results of determining the carcinogenic risk by applying the volume distribution according to the shape showed that the ECR value was less than 10^−6^ regardless of the shape, indicating that the impact on human health was not significant. Meanwhile, the ECR value calculated according to each shape had a difference of up to 39.5 times, indicating that it is still important to realistically consider the volume of MP in the risk characterization.

**Figure 4:**
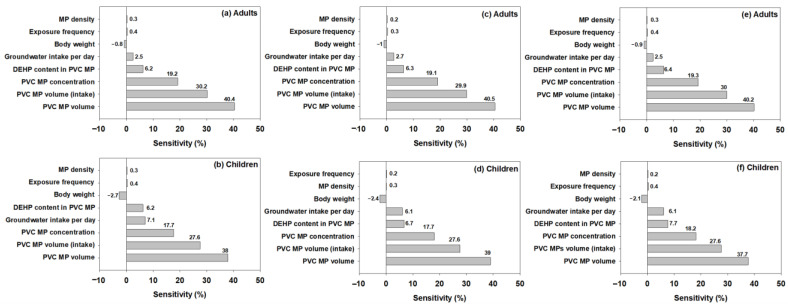
Sensitivity analysis for each parameter of human health risk assessment for each MP shape. MP volume significantly affects the assessment (MP volume sensitivity was 40.4% and 38% in the fragment (**a**,**b**), 40.5% and 39% in the fiber (**c**,**d**), and 40.2% and 37.7% in the film (**e**,**f**) shapes). The analysis results were obtained through a Monte Carlo simulation that was repeated 50,000 times.

Sensitivity analysis also showed that MP volume exhibited the highest sensitivity in groundwater for all fragment, fiber, and film shapes (Figure 4). These findings highlight the importance of MP volume in risk characterization. Since MP volume varies significantly across different shapes, the sensitivity analysis underscores the necessity of considering shape-specific volume distributions when conducting human health risk assessments. The Monte Carlo simulation results show that human health risk differs based on the shape of MPs, and accounting for the diversity of MP shapes during human health risk assessment may be necessary. Furthermore, the continuous and cumulative probability distributions of MP volumes (Figure S1) reveal that even within the same size range (20–5000 μm), significant variations in volume distributions exist across shapes. Such differences influence the DEHP exposure concentration (Cw) and subsequently affect carcinogenic risk values. Therefore, the results underscore that MP shape and its associated volume distributions are critical parameters to be considered in probabilistic human health risk assessments.

## 4. Conclusions

This study performed a probabilistic human health risk assessment using a Monte Carlo simulation and sensitivity analysis to investigate the impact of DEHP in the human body due to the intake of groundwater contaminated with PVC MPs. The impact of the diversity of PVC MP particle properties (volume by shape) on the uncertainty of human health risk assessment through ingestion was also investigated. For all shapes (fragment, fiber, and film), the human health risk assessment for DEHP in MPs showed that ECR was below 10^−6^ at 95% cumulative probability. In addition, a sensitivity analysis was performed to ascertain the significant parameters that impact the rate of carcinogenic and non-carcinogenic risks based on the route of intake of DEHP-containing MPs in the groundwater. Sensitivity analysis showed that the parameter that most affected human health risk was MP volume, which is dependent on MP shape. Therefore, illustrating that considering the shape (morphology) of MPs may lead to a more accurate human health risk assessment that could reflect the uncertainty of the diverse MP particle properties during the human health risk assessment for MPs in groundwater. Future studies should strive to consider microplastic characteristics along continuous scales, including complex properties such as aggregation, biofilm formation, or chemical composition. Incorporating such data into risk models could significantly enhance the understanding of microplastic impacts on human health.

## Figures and Tables

**Table 1:** Monte Carlo simulation parameters used to estimate di(2-ethylhexyl) phthalate (DEHP) exposure concentration.

Factors		Unit	Value	Distribution	Reference
ADD	Daily average exposure	mg/kg-day	Values calculated using Equation (8)		
HQ	Risk rate	unitless	Values calculated using Equation (7)		
ECR	ECR	unitless	Values calculated using Equation (6)		
RfD_0_	Reference dose for chronic oral exposure	mg/kg-day	0.02	Point	[51]
Sf_0_	oral slope factor	(mg/kg-day)^−1^	0.014		
C_w_	Groundwater DEHP exposure concentration	mg/L	Values calculated using C_mp_, V, ρ, R, and L of MPs (Equation (1)–(3))		-
C_w1_	Concentration of DEHP leached from MPs into groundwater	mg/L			
C_w2_	Concentration of DEHP leached from MPs into the human body (gastrointestinal fluids)	mg/L			
L_1_	Proportion of DEHP leached from MPs into groundwater based on MPs weight	%	0.0013	Point	-
L_2_	Proportion of DEHP leached from MPs into the human body based on MPs weight	%	0.25		[50]
CR_w_	Groundwater intake per day	L/day	Adults: 1.95, 0.64	Normal	[52]
			Children: 1.25, 0.57		
EF	Exposure frequency	days/year	min: 180	Triangular	
			mode: 345		
			max: 365		
ED	Exposure duration	years	Adults: 25	Point	-
			Children: 6		
BW	Body weight	kg	Adults: 70, 14	Normal	[53]
			Children: 16.67, 5.987		
AT	Average time	days	Adults: 9125Children: 2190	Point	-
C_MP_	MPs concentration	particles/L	Min: 0	Triangular	-
			Mode: 0		
			Max: 79.23		
ρ	MPs density	mg/cm^3^	1.10–1.58	Triangular	[6]
D	MPs size	μm	Values calculated using Equation (4)		
V	MPs volume	μm^3^/particles	Values calculated using Equation (4)		
R	DEHP content in MPs	w/w%	Mode: 0.29 Scale: 0.08	Minimum extreme	-

## Data Availability

The original contributions presented in this study are included in the article/Supplementary Material. Further inquiries can be directed to the corresponding author.

## Supplementary Materials

The following supporting information can be downloaded at: https://www.mdpi.com/article/10.3390/toxics13020105/s1, Table S1: Major solute concentration in the ‘artificial groundwater’ solution used in the di(2-ethylhexyl) phthalate (DEHP) leaching experiments; Table S2: Gas chromatography-mass spectrometry conditions for Di(2-ethylhexyl) phthalate (DEHP) Analysis; Table S3: Polyvinyl chloride (PVC) MP concentration surveyed at each sampling site; Table S4: Length:width:height (L:W:H) ratio for each microplastic shape used for MPs volume calculation. After Length of MPs of 20–5000 um was applied, the volume of MP was calculated according to the upper and lower limits of L:W:H; Table S5: Di(2-ethylhexyl) phthalate (DEHP) content in diverse PVC products. Figure S1: Continuous and cumulative probability distribution by MPs particle size (a, b), and continuous and cumulative probability distributions of volumes according to MPs particle size and shape (fragment, fiber, and film) determined using power functions.
