# Supplementary material for: Significance of Morphology in Characterizing Human Health Risk from di(2-ethylhexyl) Phthalate in Polyvinyl Chloride Microplastics in Groundwater
Source: Toxics. 2025 Jan 28;13(2):105. doi: 10.3390/toxics13020105 (PMC11860201; doi:10.3390/toxics13020105)
Supplement: Supplementary file 1 [file toxics-13-00105-s001.zip › toxics-3394062-supplementary.pdf]

Supplement

Significance of morphology in characterizing human health risk from di(2-ethylhexyl) phthalate in polyvinyl chloride microplastics in groundwater

Ki-Han Song[1], Sang-Gyu Yoon[2], Jin-Yong Lee[3] and Jinsung An[2,4,*]

[1]Department of Civil Engineering, Seoul National University of Science and Technology, Seoul 01811, South Korea

[2]Department of Smart City Engineering, Hanyang University ERICA, Ansan 15588, South Korea

[3]Department of Geology, Kangwon National University, Chuncheon 24341, South Korea

[4]Department of Civil & Environmental Engineering, Hanyang University ERICA, Ansan 15588, South Korea

[*] Correspondence: jsan86@hanyang.ac.kr; Tel: +82-31-400-5146.

Number of Tables: 5

Number of Figures: 1

Table S1. Major solute concentration in the 'artificial groundwater' solution used in the di(2-ethylhexyl) phthalate (DEHP) leaching experiments

Reagent	Amount of reagent
KH_2PO_4	0.30 mg/L
KNO_3	0.05 mg/L
$MgCl_2$ $6H_2O$	53.81 mg/L
$MgSO_4$	5.94 mg/L
K_2CO_3	2.04 mg/L
$MgCO_3$	34.58 mg/L
$CaCl_2$	87.12 mg/L
$NaHCO_3$	299.59 mg/L
**Characteristics**	**Value**
pH	7.7
Ionic strength	7.3 mM

Table S2. Gas chromatography-mass spectrometry conditions for di(2-ethylhexyl) phthalate (DEHP) analysis

Gas chromatograph	
Column	DB-5MS<br>(length: 30 m, diameter: 0.25 mm, film: μm)
carrier gas	He (1.0 mL/min)
Inlet temperature	280℃
Detector temperature	300℃
Oven temperature program	Step 1. 120℃<br>Step 2. Increase at 20℃/min<br>Step 3. 280℃ (hold for 2 min)<br>Step 4. Increase at 20℃/min<br>Step 5. 300℃ (hold for 5min)
Mass spectrometry	
DEHP	molecular weight: 390.55 mg/mol
Mass to charge ratio	149 m/z
Electron impact	35 eV – 70 eV

Table S3. Polyvinyl chloride (PVC) MP concentration surveyed at each sampling site [7-9]

Location	Groundwater sampling point (n)	MPs concentration (particles/L)
Donghae and Samcheok (May)	9	9.71 ± 26.14
Donghae and Samcheok (Aug.)	10	3.71 ± 11.12
Yanggu	14	3.09 ± 5.52
East Jeju	13	0.34 ± 1.23
West Jeju	8	0 ± 0

* Microplastic concentrations in the southwestern region of Jeju Island were generally lower than those in the northeastern region, and PVC was not detected at certain sampling points. This is likely due to the predominant use of PE and PP in agricultural activities in the southwestern region, with minimal use of PVC. Additionally, lower rainfall and reduced soil permeability in this region may have limited the infiltration of microplastics into the groundwater.

Table S4. Length:width:height (L:W:H) ratio for each microplastic shape used for MPs volume calculation. After Length of MPs of 20-5000 um was applied, the volume of MP was calculated according to the upper and lower limits of L:W:H [10].

Shape		Ratio (L:W:H)	
		Upper limit	Lower limit
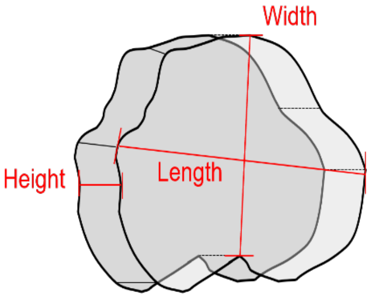	Fragment	1:1:1	1:0.1:0.01
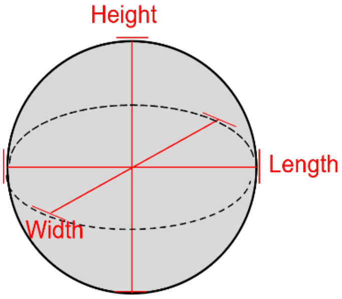	Sphere	1:1:1	1:0.6:0.36
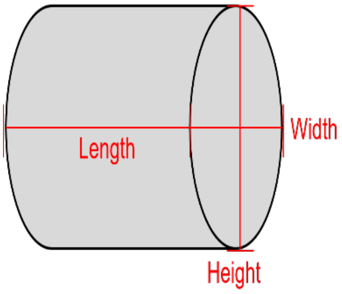	Fiber	1:0.5:0.5	1:0.001:0.001
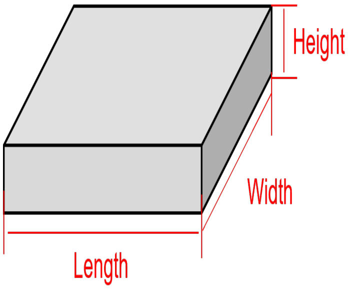	Film	1:1:0.1	1:0.1:0.01

Table S5. Di(2-ethylhexyl) phthalate (DEHP) content in diverse PVC products

Product	Content (%)	Reference
Plastic film[a]	34.1	In this study
CRM 113-03-006	0.0999	[1]
plastic film	22.4	[2]
	29.1	[3]
	24.1	
mulching film	38	[4]
	33	
	35	[5]
Tubing for transfusion	27.5	
Tubing for infusion	27	
Quadrupole bag	24	
Transfer bag	23	[6]
Dialysis tubing	22	
Dialysis bag	18	
Sag-M transfer bag	5	

a: PVC plastic film was provided by the Environmental Chemistry Laboratory of Korea University (South Korea).

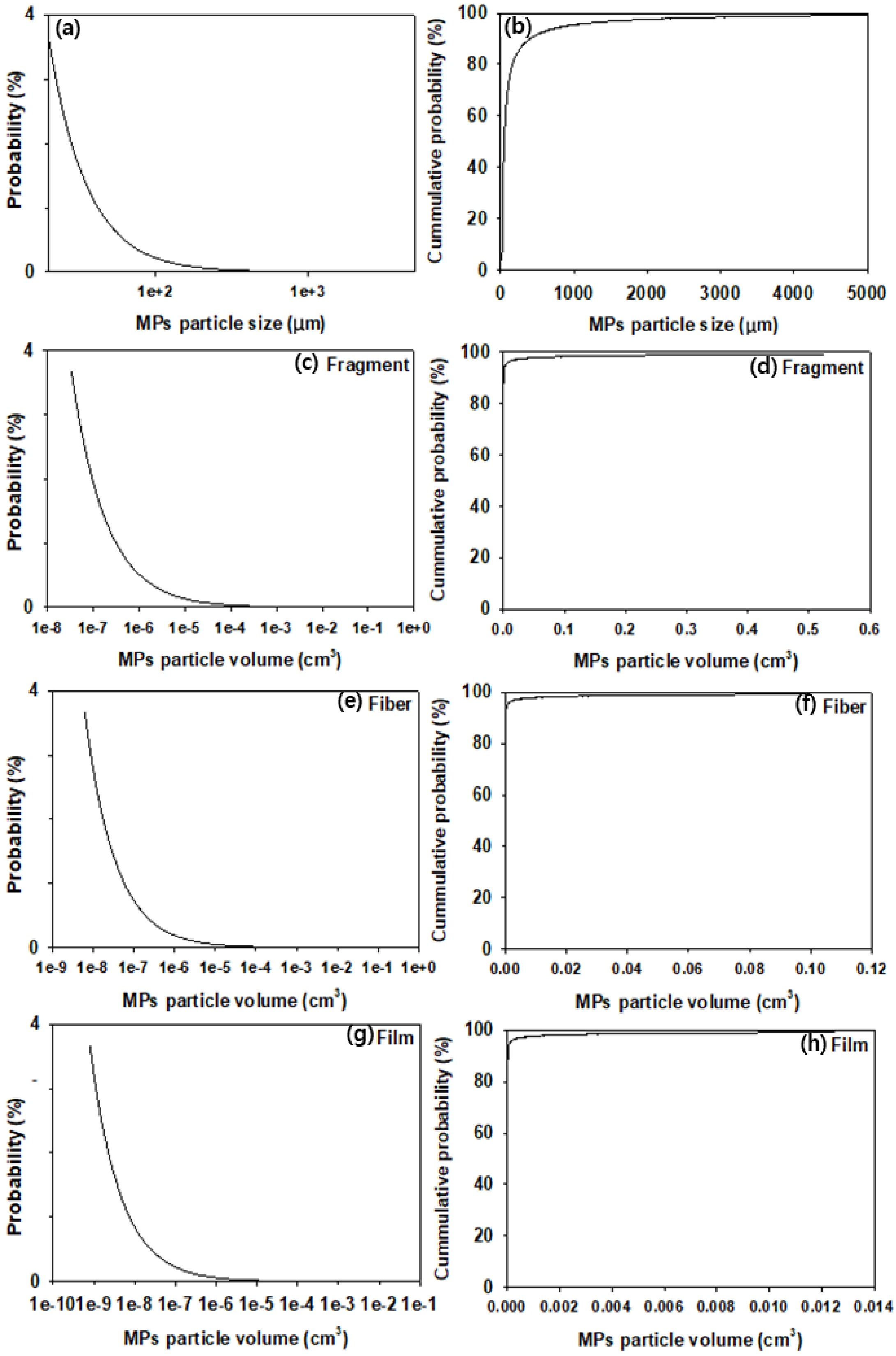

Figure S1. Continuous and cumulative probability distribution by MPs particle size (a, b), and continuous and cumulative probability distributions of volumes according to MPs particle size and shape (fragment (c, d), fiber (e, f), and film (g, h) determined using power functions.

1. Mohamed, D.F.M.S.; Kim, D.Y.; An, J.; Kim, M.; Chun, S.-H.; Kwon, J.-H. Simplified Unified BARGE Method to Assess Migration of Phthalate Esters in Ingested PVC Consumer Products. *Int J Environ Res Public Health* 2023, 20, 1907.
2. Fu, X.; Du, Q. Uptake of di-(2-ethylhexyl) phthalate of vegetables from plastic film greenhouses. *J Agric Food Chem* 2011, *59*, 11585-11588.
3. Wang, Y.; Wang, F.; Xiang, L.; Gu, C.; Redmile-Gordon, M.; Sheng, H.; Wang, Z.; Fu, Y.; Bian, Y.; Jiang, X. Risk Assessment of Agricultural Plastic Films Based on Release Kinetics of Phthalate Acid Esters. *Environ Sci Technol* 2021, *55*, 3676-3685.
4. Henkel, C.; Hüffer, T.; Hofmann, T. Polyvinyl Chloride Microplastics Leach Phthalates into the Aquatic Environment over Decades. *Environ Sci Technol* 2022, *56*, 14507-14516.
5. Henkel, C.; Hüffer, T.; Hofmann, T. The leaching of phthalates from PVC can be determined with an infinite sink approach. *MethodsX* 2019, *6*, 2729-2734.
6. Andjelković, T.; Bogdanović, D.; Kostić, I.; Kocić, G.; Nikolić, G.; Pavlović, R. Phthalates leaching from plastic food and pharmaceutical contact materials by FTIR and GC-MS. *Environ Sci Pollut Res* 2021, *28*, 31380-31390.
7. Jeong, E., Kim, Y.-I., Lee, J.-Y., Raza, M., 2023. Microplastic contamination in groundwater of rural area, eastern part of Korea. Science of The Total Environment. 895. 165006.
8. Kim, Y.-I., Jeong, E., Lee, J.-Y., Chia, R.W., Raza, M., 2023. Microplastic contamination in groundwater on volcanic Jeju Island of Korea. Environmental Research. 226. 115682.
9. Cha, J., Lee, J.-Y., Chia, R.W., 2023. Microplastics contamination and characteristics of agricultural groundwater in Haean Basin of Korea. Science of The Total Environment. 864. 161027.
10. Kooi, M.; Koelmans, A.A. Simplifying Microplastic via Continuous Probability Distributions for Size, Shape, and Density. *Environ Sci Technol Lett* 2019, 6, 551-557.
